# Flattening the Curve of COVID-19 Vaccine Rejection—An International Overview

**DOI:** 10.3390/vaccines9010044

**Published:** 2021-01-13

**Authors:** Wojciech Feleszko, Piotr Lewulis, Adam Czarnecki, Paweł Waszkiewicz

**Affiliations:** 1 Department of Pediatric Pulmonology and Allergy, Medical University of Warsaw, 02-091 Warsaw, Poland; 2 Department of Criminalistics, University of Warsaw, 00-927 Warsaw, Poland; p.lewulis@wpia.uw.edu.pl (P.L.); p.waszkiewicz@uw.edu.pl (P.W.); 3 ARC Rynek i Opinia, Market Research Institute, 01-627 Warsaw, Poland; adam.czarnecki@arc.com.pl

**Keywords:** COVID-19, vaccines, vaccine hesitancy, herd immunity

## Abstract

Background: If globally implemented, a safe coronavirus disease 2019 (COVID-19) vaccination program will have broad clinical and socioeconomic benefits. However, individuals who anticipate that the coronavirus vaccine will bring life back to normality may be disappointed, due to the emerging antivaccination attitude within the general population. Methods: We surveyed a sample of adult Polish citizens (*n* = 1066), and compared it with the data on international COVID-19 vaccine reluctance. Results: In 20 national surveys, the vaccine averseness for the anticipated COVID-19 vaccine varied from meager (2–6% China) to very high (43%, Czech Republic, and 44%, Turkey) and in most countries was much higher than regular vaccination reluctance, which varies between 3% (Egypt) and 55% (Russia). Conclusions: These results suggest that a 67% herd immunity may be possible only if mandatory preventive vaccination programs start early and are combined with coordinated education efforts supported by legislative power and social campaigns.

## 1. Introduction

As the coronavirus disease 2019 (COVID-19) spreads unprecedented socioeconomic chaos, many vaccine development projects have been launched simultaneously in various countries [1]. Recently, the United States authorities have approved two coronavirus vaccines, and the approval of a third is pending. Existing reports indicate their effectiveness at around 95% [2,3]. However, as we expect the development of several other vaccines in the upcoming months, societies worldwide are likely to be deluged with questions and public arguments about the vaccines and their safety [4].

What may be puzzling, especially for nonscientific audiences, is that some projects have been discontinued or put on hold due to safety reasons, while others have been declared safe and effective by Russian and Chinese officials, despite not having passed rigorous efficacy trials [5,6]. At the same time, researchers and policymakers must consider how to deal with challenges that are unrelated to the vaccine candidates themselves. These include vaccine hesitancy, weariness with current public health restrictions, and the staggering logistics of vaccinating the world’s population.

The high pace of work on vaccine development worldwide, and the potentially premature implementation of a poorly tested vaccine, may fuel—rightly or not—the already existing vaccination fears. Hence, any antiSARS-CoV-2 (Severe Acute Respiratory Syndrome Coronavirus 2) vaccination program is put at risk by the already worrying levels of public acceptability of immunization, as it has already been pointed out in previous research [7]. Unfortunately, vaccination hesitancy, especially in the context of a COVID-19 vaccine, is a real global phenomenon, and anti-vaccination movements are increasingly influential [1,8].

Collective herd-acquired immunity is based on the mass implementation of vaccination programs, so reluctance to participate in them may seriously undermine the efforts to protect societies against COVID-19 in the near future [8]. Serious concerns may be raised about public participation in vaccination programs once the COVID-19 vaccine is finally available, particularly taking into account the rising numbers of adults questioning the validity of preventive vaccination programs in general [9]. Therefore, we aimed to estimate confidence in vaccination in both national and international contexts, and check its correlation with the willingness to participate in the COVID-19 vaccination program in the future.

## 2. Materials and Methods

The primary study goal was to provide knowledge about the attitude towards hypothetical COVID-19 vaccination in Polish society, and identify factors that potentially influence decisions. To that end, we conducted an opinion survey in Poland through an online omnibus survey tool on 2–9 June 2020. Quota sampling and statistical weighting was employed to make the sample representative of Poland’s offline population aged 18–65, by gender, age, region, and city size. The survey was completed on https://epanel.pl/ (N = 1066), in an online opt-in panel operated by the ARC Rynek i Opinia Independent Research Institute. The Cadas software was applied for online self-completed questionnaires (CADAS Software Sp. z o.o., Warsaw, Poland), and then the randomization of items in multichoice questions was applied. Weighted response frequencies were calculated using SPSS software. Statistical analysis was completed, and any subgroup differences included are statistically significant at a 95% confidence interval. The data file provided by the Research Institute contained no personally identifying information.

An additional study goal was to allow for a comparative perspective on the Polish research survey results, and provide a general international overview of COVID-19 vaccination hesitancy. For this purpose, we collected the available data on COVID-19 vaccination willingness in other countries using online open source data-gathering methods. Subsequently, we put all the information gathered into the greater context of prepandemic data, abstract attitudes towards vaccination effectiveness, and safety in general.

Therefore, the study consisted of two stages: data collection and analysis.

### 2.1. Stage (1)

The first stage involved an online search and data collection from all available opinion polls on vaccination against COVID-19, conducted in various countries. The study’s online sources included openly available scientific papers, research and survey reports, official documents, and news media presenting information about opinion polls on personal attitudes towards hypothetical COVID-19 vaccination [7,10,11,12,13,14,15,16,17,18,19,20,21,22,23,24,25,26,27,28]. Any given opinion-poll results found online were analyzed if the poll had been performed by a reliable and trusted entity (i.e., an academic unit or a professional survey research company) on a representative sample of the general adult population between March and July, 2020. The polls must have contained a closed question (preferably a ‘*yes*’ or ‘*no*’ question) about one’s hypothetical decision about COVID-19 vaccination (e.g., ‘*if a new Coronavirus vaccine became publicly available, I would be willing to be vaccinated*’ or similar, and phrased differently according to the relevant national language). Only one research survey per country was taken into account. Surveys performed on nonrepresentative samples, such as various internet opinion polls available on news sites, were excluded from the study. The search was conducted between 8 and 10 June 2020, using fundamental online data-gathering tools: search engines (google.com and google.scholar.com), and an automatic translation tool (google.translate.com). The search was conducted in a total of 32 national languages (in alphabetical order: Arabic, Belarusian, Bulgarian, Czech, Danish, Dutch, English, Estonian, Finnish, Flemish, French, Georgian, German, Hindi, Hungarian, Icelandic, Italian, Japanese, Kazakh, Korean, Latvian, Lithuanian, Norwegian, Portuguese, Romanian, Russian, Slovakian, Spanish, Swedish, Thai, Turkish, and Ukrainian). The internet searches were conducted using google.translate.com to specify search terms and keywords in all the national languages except for English, French, German, Italian, Portuguese, and Turkish, in which the search terms were defined directly. Various keywords and search parameters simultaneously used in the study were specified to enable relevant data to be found (e.g., ‘COVID-19 vaccination acceptance + opinion poll’, or ‘*would you vaccinate against coronavirus + opinion survey*’, and others similar but differently phrased to maximize search coverage). The list of languages was sufficient to seek information from countries from all the world’s regions (Europe, Africa, the Americas, Asia and Oceania). As a result, we found surveys meeting the specified criteria from 20 different jurisdictions. For certain regions, we found no reliable data available at the time of the study; what may be considered surprising at that point of the pandemic is that there was no such data for countries facing the deadly wave of COVID-19, like Brazil, India, Japan, or Korea.

A relevant reference list of sources is incorporated into the reference section. The data collected were translated and entered into the data table as representing positive or negative social attitudes towards COVID-19 vaccination during the analysis stage. Uncertain answers to the survey question, such as ‘*rather yes*’ and ‘*rather no*’, were extrapolated to ‘*yes*’ and ‘*no*’, respectively.

### 2.2. Stage (2)

The collected data were subject to comparative analysis in the previous study on the general public’s perception of vaccination effectiveness and safety in the same countries. Such comparison aimed to estimate the scope of possible discrepancies between the number of people declaring the will to vaccinate against COVID-19 and the number of those declaring confidence in vaccination effectiveness and safety in general.

The outbreak of COVID-19 includes a potent psychological factor that might influence the public perception of vaccines and vaccination. However, even if individual views on the subject have changed, the data from 2018 are useful to illustrate the minimum potential level of confidence in vaccines. In other words, they are more reliable in reflecting rational or undistorted public views on the subject, which of course may have changed due to recent events.

The 2018 Wellcome Foundation Global Monitor study report provided data on the overall general approach to vaccination effectiveness and safety [9]. The study allows for a reliable intersubjective results comparison, as it uses a single methodology and a uniform questionnaire collected from all countries in a defined timeframe (April–December, 2018). The study is from 2018—relatively shortly before the COVID-19 pandemic. We consider this an added advantage of the study because intense aversive feelings of fear and uncertainty in crisis times stimulate tendencies to engage in conspiracy narratives [29].

## 3. Results

### 3.1. National Survey Results

A survey on a representative sample of adult Polish citizens (*n* = 1066, conducted on 2–9 June 2020) showed that 28% of adults in Poland would not vaccinate against SARS-CoV-2 if the vaccine became available. Alarmingly, a majority (51%) of the reluctant respondents indicated that their minds would neither be changed by information regarding vaccine safety and efficacy, nor if they were threatened with hefty fines. Significantly fewer respondents (37%) supported COVID-19 vaccinations specifically than supported childhood vaccinations in Poland in general (78% in 2018) (Table 1).

There are some significant differences in attitudes toward the COVID-19 vaccination, depending on gender and age. Female respondents and those in middle adulthood groups (25–44 years) are more reluctant to undergo vaccination than male respondents, young adults (18–24), and older adults (46–65) (Table 1).

All the respondents who answered that they ‘*do not plan to vaccinate if the COVID-19 vaccine is available*’ (N = 301) were confronted with a list of eight different hypothetical reasons and asked if any of them would convince them to change their minds and vaccinate. The list of presented reasons included: presentation of scientific research on vaccine safety (i); statements of experts—doctors, scientists, etc., (ii); vaccination was recommended by a family doctor (iii); someone from my loved ones/family/friends was vaccinated (iv); a public figure, whom I trust and respect, was vaccinated (v); low cost/no cost of the vaccine (vi); high fines for not vaccinating yourself or your child (e.g., Polish Zloty (PLN) 5000 ≈ €1.500) (vii); and that it would not be possible to enter some countries without a vaccination certificate (viii). Against such a choice, most (51%) answered that none of the presented reasons would change their decision. Again, there were visible significant differences in attitudes toward hypothetical reasons correlating to the gender and the age of respondents. Especially the latter played a significant role. The younger respondents were the group most often declaring to rely on scientific research on vaccine safety and family doctors’ recommendations. They also declared almost four times more often than older adults (27% vs. 7%) that high fines would convince them to vaccinate against COVID-19 (Table 2).

### 3.2. International Vaccine Hesitancy Results

**Figure 1:**
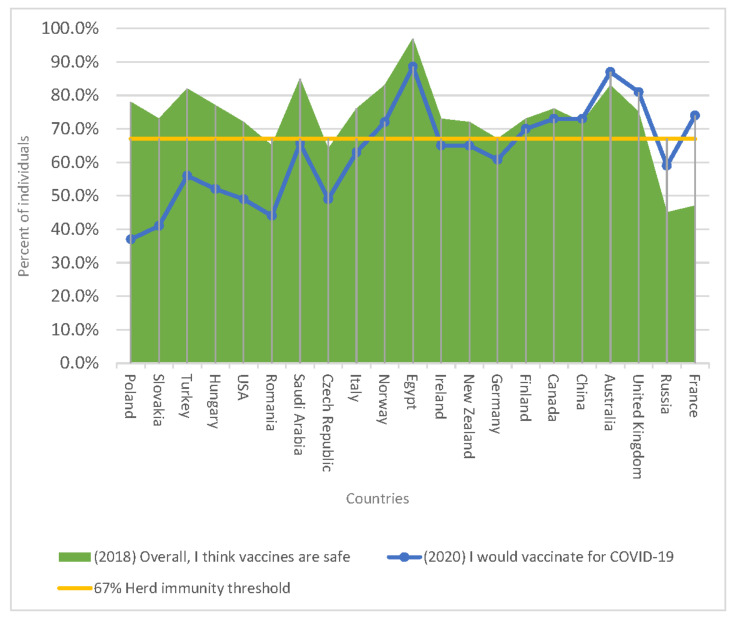
An international overview and comparison of COVID-19 vaccination acceptance levels and attitudes towards vaccination in general, with a graphic representation in the context of the herd-immunity vaccination threshold (yellow horizontal line).

To validate these data from a broader perspective, we compared the results with the available data from other countries. A systematic search of nationally representative and methodologically sound surveys identified a total of 20 countries (Table 3). The vaccine hesitancy for the hypothetical-yet-anticipated (as assessed in June, 2020) COVID-19 vaccine varied from very low (2–6% China) [12] to very high (43%, Czech Republic, and 44%, Turkey) [13,26]. The level of unwillingness to vaccinate against COVID-19 is in most countries much higher than the regular vaccination reluctance, which varies between 3% (Egypt) and 55% (Russia) [14,23]. The reluctance to vaccinate against COVID-19, however, is generally not reflected in abstractly expressed views on the efficacy or safety of vaccination. A graphical representation of the data (Figure 1) illustrates the scale of the discrepancies between abstractly expressed confidence in vaccination and the willingness to vaccinate against COVID-19.

## 4. Discussion

According to the survey results, there is considerable societal opposition to the COVID-19 vaccination, which is currently available in most countries. In this study, we gathered international data from 21 countries and provided evidence that vaccination hesitancy is a global phenomenon, even in the wake of the COVID-19 pandemic.

Fueled by widespread misinformation, vaccine hesitancy (a ‘delay in acceptance or refusal of vaccines despite availability of vaccination services’ [2]) has quickly become one of the most important global health issues of our time. The statistics speak for themselves. The most important findings resulting from our research are that we have identified early a high COVID-19 vaccination reluctance. In this context, it is essential to prepare early the grounds for future vaccination programs through education and legislative action [30]. Mandatory vaccination should be seriously considered in particular in some occupations that are associated with a heightened risk of contracting COVID-19: healthcare workers (e.g., dental hygienists, family and general practitioners, and nurses), transportation personnel (e.g., flight attendants and school bus drivers), kindergarten and school teachers, firefighters, and restaurant personnel [31]. Additionally, the older adults who are reluctant towards a COVID-19 vaccine seem to be the hardest to convince, at least according to their declarations (Table 2). Therefore, a legislative action and the creation of sound and coherent standard, global, public policies should precede the availability of an effective and safe COVID-19 vaccine, in the view of our results.

Most of the public opinion surveys that we analyzed in this study were conducted in May/June, 2020, which may be, to some extent, a limitation of those findings. Another limitation is that various national surveys used different methodologies, including the Computer-Assisted Web Interview (CAWI) technique, which often excludes the oldest (age 65 and older) citizens. Since they are in the high-risk group, this may reveal a serious flaw.

A similar international survey was conducted by the French market research and consulting firm Ipsos Group (France). This study (IPSOS study), was conducted between 24 July and 7 August 2020, in 27 countries; however, it corroborates the presented findings [30]. Both willingness and reluctance to vaccinate are increasing in individual societies in line with previously expressed views. There are a couple of differences between the IPSOS study and the presented survey. The main one is that the IPSOS study was commissioned by one organization with all its strengths and limitations, while our report consists of data from 21 independent surveys. Only one of these was carried out by the authors themselves. It reduces the researcher bias, which could exert an influence on the final results.

In some societies, there is also a reluctance to multinational companies, such as IPSOS. It may lead to the declining participation of some groups of possible respondents. On the other hand, a responsible and experienced company such as IPSOS may organize such a global survey with consideration of those limitations.

## 5. Conclusions

High levels of COVID-19 vaccination hesitancy may severely limit the success of currently rolling vaccination programs’ effectiveness, particularly when fueled by ‘big pharma’ and other conspiracy narratives popular on social media [31]. According to current estimates, herd immunity benefits are achievable if 65%–70% of the population is vaccinated [32]. The high share of the population unwilling to vaccinate, combined with the number of people unable to receive the COVID-19 vaccine (e.g., for medical reasons), suggests that herd immunity may be out of reach. Some vaccination programs in the past effectively eradicated certain deadly diseases; however, this success was only possible thanks to the combination of mandatory preventive vaccination programs with coordinated education efforts [33].

Opinion surveys and research to date have looked at the reasons for which individuals are reluctant to vaccinate, and there is still much to be found on this point. Research on vaccine hesitancy assumes that it is vital to understand the reasons behind individual attitudes to tailor the communication and immunization programs accordingly [34]. Therefore, an investigation into specific COVID-19 vaccination willingness and reluctance may be crucial to inform future legislative and educational actions better. Therefore, future opinion research should focus on why people do and do not want to get vaccinated against COVID-19. The knowledge about these reasons may help us to design better solutions to increase immunization coverage in countries most affected by the vaccination hesitancy problem.

## Figures and Tables

**Table 1:** If a vaccine against coronavirus disease 2019 (COVID-19) is available and safe, do you plan to vaccinate?

Answer Provided	Total	Gender		Age			
		Female	Male	18–24	25–34	35–44	46–65
Yes	37%	31%	43%	43%	32%	33%	41%
No	28%	31%	25%	25%	36%	30%	24%
I do not know/it is difficult to answer	34%	38%	31%	32%	33%	37%	35%
Weighted base	1066	535	531	125	243	254	445

**Table 2:** Would any of the listed reasons convince you to vaccinate against COVID-19?

Hypothetical Reasons Convincing to Vaccinate	Total	Gender		Age			
		Female	Male	18–24	25–34	35–44	46–65
Presentation of scientific research on vaccine safety	17%	17%	18%	20%	18%	21%	14%
Statements of experts—doctors, scientists, etc.	9%	9%	8%	6%	13%	7%	7%
Vaccination was recommended by a family doctor	6%	7%	6%	13%	7%	7%	7%
Someone from my loved ones/family/friends was vaccinated	6%	8%	3%	10%	6%	7%	3%
Apublic figure, whom I trust and respect, was vaccinated	7%	8%	6%	7%	7%	7%	8%
Low cost/no cost of the vaccine	6%	4%	8%	3%	7%	6%	6%
High fines for not vaccinating yourself or your child (e.g., PLN 5000 ≈ €1.500)	10%	12%	8%	27%	10%	10%	7%
It would not be possible to enter some countries without a vaccination certificate	11%	9%	13%	14%	17%	6%	8%
None of the above	51%	51%	51%	37%	43%	53%	59%
Weighted base	301	166	135	31	87	76	107

**Table 3:** An international overview and comparison of COVID-19 vaccination acceptance levels and attitudes towards vaccination in general.

Country	2018			2020		
	Overall, I Think Vaccines Are Safe	Overall, I Think Vaccines Are Effective	N	I Would Vaccinate for COVID-19	I Would Not Vaccinate for COVID-19	N
Egypt	97.0%	96.0%	(*n* = 1000)	88.6%	7.2%	(*n* = 559)
Australia	83.0%	91.0%	(*n* = 1003)	87.0%	7.0%	(*n* = 987)
United Kingdom	75.0%	86.0%	(*n* = 1000)	81.0%	4.0%	(*n* = 2025)
France	47.0%	68.0%	(*n* = 1000)	74.0%	26.0%	(*n* = 1012)
China	72.0%	79.0%	(*n* = 3649)	73.0%	2.6%	(*n* = 189)
Canada	76.0%	83.0%	(*n* = 1012)	73.0%	27.0%	(*n* = 1000)
Norway	83.0%	93.0%	(*n* = 1000)	72.0%	n/a	(*n* = 1172)
Finland	73.0%	83.0%	(*n* = 1000)	70.0%	22.0%	(*n* = 1000)
Saudi Arabia	85.0%	83.0%	(*n* = 1016)	65.7%	7.0%	(*n* = 992)
Ireland	73.0%	86.0%	(*n* = 1000)	65.0%	9.0%	(*n* = 1000)
New Zealand	72.0%	82.0%	(*n* = 1002)	65.0%	16.0%	(*n* = 605)
Italy	76.0%	84.0%	(*n* = 1000)	63.0%	21.0%	(*n* = 1325)
Germany	67.0%	83.0%	(*n* = 1000)	60.8%	n/a	(*n* = 972)
Russia	45.0%	62.0%	(*n* = 2000)	59.0%	35.0%	(*n* = 1600)
Turkey	82.0%	87.0%	(*n* = 1000)	56.0%	44.0%	(*n* = 1537)
Hungary	77.0%	78.0%	(*n* = 1000)	52.0%	23.0%	(*n* = 1000)
USA	72.0%	84.0%	(*n* = 1006)	49.0%	20.0%	(*n* = 1056)
Czech Republic	64.0%	76.0%	(*n* = 1000)	49.0%	43.0%	(*n* = 1007)
Romania	65.0%	75.0%	(*n* = 1002)	44.0%	33.0%	(*n* = 1027)
Slovakia	73.0%	90.0%	(*n* = 1000)	41.0%	28.0%	(*n* = 1000)
Poland	78.0%	84.0%	(*n* = 1000)	37.0%	28.0%	(*n* = 1066)

Scales of red and green reflect percentage data. Color saturation increases with percentage.

## Data Availability

Part of the data available in a publicly accessible repositories (see list of references). The survey data available on request due to privacy.
